# Transgenerational effects in DNA methylation, genotoxicity and reproductive phenotype by chronic arsenic exposure

**DOI:** 10.1038/s41598-021-87677-y

**Published:** 2021-04-15

**Authors:** Lydia Enith Nava-Rivera, Nadia Denys Betancourt-Martínez, Rodrigo Lozoya-Martínez, Pilar Carranza-Rosales, Nancy Elena Guzmán-Delgado, Irma Edith Carranza-Torres, Hector Delgado-Aguirre, José Omar Zambrano-Ortíz, Javier Morán-Martínez

**Affiliations:** 1 Departamento de Biología Celular y Ultraestructura, Centro de Investigación Biomédica, Facultad de Medicina, Universidad Autónoma de Coahuila Unidad Torreón, Gregorio A. García No. 198 sur. Colonia centro, Torreón, Coahuila CP 27000 México; 2 Centro de Investigación Biomédica del Noreste, Instituto Mexicano del Seguro Social, Monterrey, Nuevo León Mexico; 3 División de Investigación en Salud, Unidad Médica de Alta Especialidad, Hospital de Cardiología #34, Instituto Mexicano del Seguro Social, Monterrey, Nuevo León Mexico; 4 Laboratorio de Histocompatibilidad, Unidad Médica de Alta Especialidad (UMAE) # 71, Instituto Mexicano del Seguro Social, Torreón, Coahuila Mexico

**Keywords:** Evolution, Genetics, Molecular biology

## Abstract

An emerging concern is the influences of early life exposure to environmental toxicants on offspring characteristics in later life. Since recent evidence suggests a transgenerational transference of aberrant phenotypes from exposed-parents to non-exposed offspring related to adult-onset diseases including reproductive phenotype. The transgenerational potential of arsenic a well know genotoxic and epigenetic modifier agent has not been assessed in mammals until now. In this experimental study, we evaluated the transgenerational effects of arsenic in a rat model with chronic exposure to arsenic. Rats chronically exposed to arsenic in drinking water (1 mg As_2_O_3_/L) (F0) were mated to produce the arsenic lineage (F1, F2, and F3). The arsenic toxic effects on were evaluated over the four generations by analyzing the DNA methylation percentage, genotoxicity in WBC and physical and reproductive parameters, including sperm quality parameters and histopathological evaluation of the gonads. Chronic exposure to arsenic caused genotoxic damage (F0–F3) different methylation patterns, alterations in physical and reproductive parameters, aberrant morphology in the ovaries (F0 and F1) and testicles (F1–F3), and a decrease in the quality of sperm (F0–F3, except F2). Parental chronic arsenic exposure causes transgenerational genotoxicity and changes in global DNA methylation which might be associated with reproductive defects in rats. Combined with recent studies reveal that disturbances in the early life of an individual can affect the health of later generations.

## Introduction

Arsenic (As), an omnipresent element in the earth's crust^1^ and a knowing toxicant agent in humans, continues being a research objective since millions of people worldwide are still chronically exposed to arsenicals, mainly through drinking water, keeping it as a major public health problem^2^. An emerging concern is the consequences of As early-life exposure since there is evidence of adverse effects of gestational or even earlier exposure in parents’ life (F0 generation) to diverse environmental toxicants, can lead to adult onset disease in the offspring (F1 generation) or in a multigenerational (F2 generation) or transgenerational way (F3 and beyond)^3–5^. The mechanisms that participate in this developmental programming in which early life exposure may affect the later-life are in continuous researching, this is really important since, besides the genotoxicity caused by some environmental toxicants, these can generate epigenetics changes that also can regulate gene expression and be transgenerationally transmitted^6^. Related to arsenic, some adverse later-onset phenotypes have been identified; epidemiological studies have been related in utero arsenic exposure with an elevated risk of cancer, respiratory and cardiovascular diseases in offspring^7^. Multigenerational evidence in animal models suggest that gestational arsenic exposure increases hepatic tumor incidence from F1–F2^8^.

From the toxicological point of view the trivalent inorganic arsenic compounds (As^III^, also known as arsenite) are the most toxic due to its reactivity with sulfur-containing compounds and the generation of reactive oxygen species and nitrogen species (ROS/RNS), mechanism by which it can induce a well-known genotoxic effect, besides there is information indicating the relation of As with epigenetic modifications, suggesting the induction of adverse effects to human health, through a key mechanism of gene regulation, the DNA methylation^9–13^, which is an heritable epigenetic mechanism that occurs at position 5 of the cytosine residues (5-mC) and ensure the correct packaging of DNA and regulation of gene expression in eukaryotic cells^14^, changes in the global methylation status are related to the environment exposition being propose as a potential biomarker of diseases^15^. Arsenic could modify the methylation profiles in mammals disturbing the activity of the DNA methyltransferase (DNMT) enzymes or trough depletion of the S-adenosylmethionine (SAM) cofactor during its metabolization^16–18^. Recently, the multigenerational skin impact of arsenic through the assessment of global DNA methylation resulting in relevant changes^19^. However, the transgenerational effects caused by this metalloid has not been already explorer in mammals. Otherwise in the developmental programming research is particularly of relevance the multigenerational reproductive dysfunction following exposure to potent toxicants such as vinclozolin and methoxychlor^20, 21^. As is a knowing reproductive toxicant that generate adverse effects on the reproductive ability of males and females, as well as adverse effects on the development of offspring^22^. Various studies have found significant associations between arsenic exposure and adverse infant outcomes, such as spontaneous abortion, low birth weight, and infant mortality^23–25^. In terms of male reproductively exposure from drinking water significantly causes low sperm quality and erectile dysfunction^26, 27^, in animal models sperm count, motility and morphology were affected principally in long exposures to relatively low concentrations, besides ovarian an uterine disorders^28–32^. The transgenerational reproductive effects in *C. elegans* caused by this metalloid^33^, has already been assessed, moreover these effects may not have been evaluated in mammals. Here we investigated the transgenerational reproductive effects of As in drinking water exposition through a rodent design based in the expose populations: with an early-life and chronical parental exposure (F0 generation). In addition, the evaluation of the potential of As to cause changes in DNA methylation and genotoxicity over generations (F0–F3).

## Results

### Arsenic effects in physical parameters

Arsenic prenatal exposure is inversely associated with infant size at birth in humans^34, 35^, however in rodents there is evidence of not bodyweight alterations in pups^36, 37^, besides an evident change in organs weight such as kidney, liver, epididymis and gonads^38–40^. In this study the body weight and relative weight (% of body weight) of various organs (liver, kidneys, epididymis and gonads) were measured and compared intra-generationally between exposed groups and their age-matched control groups (Tables 1 and 2).

**Table 1:** Body weight and organ relative weight over generations in males.

Generation	n	Group	Basal body weight (g)/ height (cm)	Final weight (g)	Liver (% bw)	Kidneys (% bw)	Testicles (% bw)	Epididyms (% bw)
F0	6	Control	69.55 ± 5.35	381.88 ± 54.18	3.76 ± 0.26	0.64 ± 0.04	0.83 ± 0.08	0.44 ± 0.04
F0	6	Exposed	70.52 ± 2.97	359.32 ± 51.53	3.29 ± 0.37*	0.56 ± 0.06*	0.78 ± 0.09	0.35 ± 0.04*
F1	10	Control group offspring	6.62 ± 0.74/ 6.66 ± 0.40	363.28 ± 45.47	2.61 ± 0.24	0.31 ± 0.03	0.50 ± 0.05	0.18 ± 0.02
F1	10	Exposed group offspring	5.99 ± 1.59/ 6.43 ± 0.61	316.13 ± 40.88	3.37 ± 1.32	0.32 ± 0.06	0.51 ± 0.04	0.20 ± 0.04
F2	10	Control group lineage	6.47 ± 0.38/ 7.03 ± 0.36	382.33 ± 9.93	4.48 ± 0.14	0.39 ± 0.02	0.53 ± 0.04	0.10 ± 0.01
F2	10	Exposed group lineage	6.57 ± 0.18/ 6.57 ± 0.15*	246.70 ± 12.30*	4.04 ± 0.08*	0.38 ± 0.01	0.81 ± 0.01*	0.05 ± 0.00*
F3	10	Control group lineage	6.23 ± 0.40/ nd	340.07 ± 53.58	3.90 ± 0.17	0.33 ± 0.02	0.53 ± 0.06	0.16 ± 0.07
F3	10	Exposed group lineage	5.43 ± 0.35/ nd	232.00 ± 13.96*	3.24 ± 0.17*	0.33 ± 0.02	0.69 ± 0.01*	0.22 ± 0.02*

(*) Statistically significant differences between control and arsenic exposed group (mean ± standard deviation) compared intra-generationally (F0–F3) (P < 0.05).

*% bw* percentage of body weight, *nd* not determined.

**Table 2:** Body weight and organ relative weight over generations in females.

Generation	n	Group	Basal body weight (g)/ height (cm)	Final weight (g)	Liver (% bw)	Kidneys (% bw)	Ovaries (% bw)
F0	6	Control	74.33 ± 1.63	283.18 ± 30.15	4.75 ± 0.77	0.75 ± 0.03	0.08 ± 0.02
F0	6	Exposed	72.45 ± 2.13	248.05 ± 19.81*	3.84 ± 0.13*	0.70 ± 0.06	0.08 ± 0.01
F1	10	Control group offspring	6.45 ± 0.80/ 6.63 ± 0.30	265.80 ± 25.24	2.33 ± 0.50	0.31 ± 0.03	0.03 ± 0.01
F1	10	Exposed group offspring	5.69 ± 1.37/ 6.33 ± 0.47	243.63 ± 16.95	2.75 ± 0.58	0.32 ± 0.02	0.03 ± 0.01
F2	10	Control group lineage	6.78 ± 0.27/ 6.74 ± 0.23	217.51 ± 12.38	4.12 ± 0.21	0.43 ± 0.02	0.02 ± 0.01
F2	10	Exposed group lineage	6.32 ± 0.44/ 6.64 ± 0.21	200.35 ± 18.35*	4.40 ± 0.17	0.41 ± 0.01	0.02 ± 0.00
F3	10	Control group lineage	6.63 ± 0.15**/ nd**	274.90 ± 33.00	5.01 ± 0.85	0.36 ± 0.00	0.03 ± 0.00
F3	10	Exposed group lineage	5.73 ± 0.42**/ nd**	196.96 ± 23.89*	4.14 ± 0.36	0.36 ± 0.02	0.03 ± 0.01

(*) Statistically significant differences between control and arsenic exposed group (mean ± standard deviation) compared intra-generationally (F0-F3) (P < 0.05).

*% bw* percentage of body weight, *nd* not determined.

There was no significant change in the body weight in pups over generations. However, a decrement in final body weight across male and female generations of the arsenic lineage was observed. The final body weights of the F2 and F3 generation arsenic lineage males were decreased significantly, also in the arsenic lineage females including the F0 exposed generation. Following arsenic exposure, the liver weight in males of the F0, F2 and F3 and in females only in F0 generation were significant decreased. The kidneys weight was only significantly decreased in F0 exposed male generation. The testicles weights were significantly increased in F2 and F3 generation arsenic lineage as well epididymal weights including F0 exposed generation. There were not significant differences in ovarian weights over generations.

### Number of pups in F0–F2

There is evidence that support the association between high inorganic arsenic exposure and spontaneous abortion in humans^41^ and in rodents^42^. Here, the number of pups born was compared intra-generationally.

**Figure 1:**
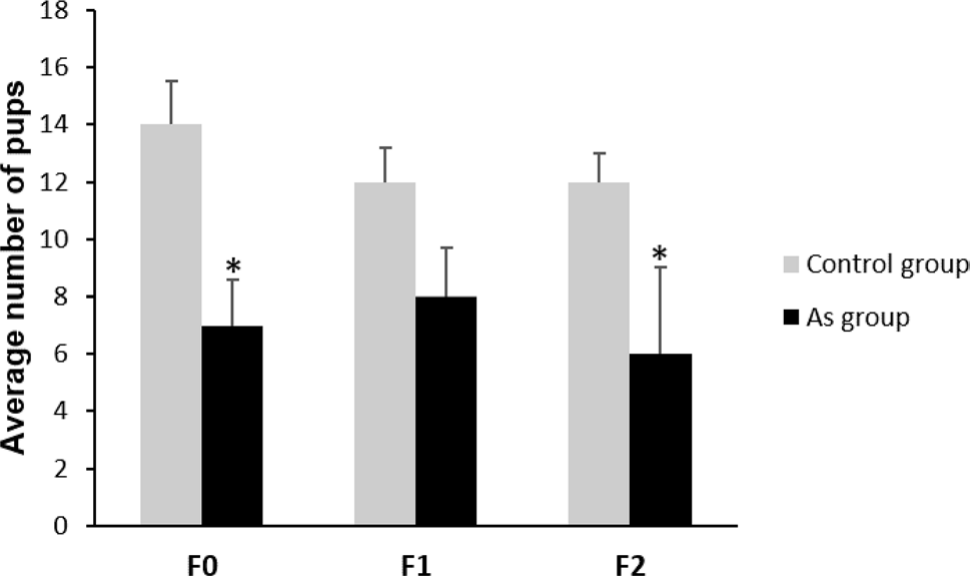
Average number of pups from F0, F1 and F2 generation. (*) statistically significant differences between control (n = 15) and arsenic exposed group (n = 15) compared intra-generationally (P < 0.05). Error bars: standard deviation.

A significant decrease in the number of pups born from the arsenic lineage was observed. In F0 generation an average of 7 pups born in the exposed group compared to 14 pups in the control group. In F2 generation, 6 pups from the arsenic lineage compared to 12 pups in the control lineage group. A decrease was also observed in F1 generation arsenic lineage however, this was not statistically significant (Fig. 1). The mortality rate was 0, in addition to the fact that none of the puppies presented any type of evident malformation after the physical examination.

### Sperm quality

Inorganic arsenic exposure is associated with reproductive dysfunctions including sperm motility, viability, morphology and count including epididymal sperm count mediated trough alterations in spermatogenesis and endocrine disruptions in humans, rats and mice^43–45^. Here, sperm quality parameters were evaluated and compared transgenerationally.

Sperm parameters were significantly decreased in the arsenic linage compared to control lineage rats, except in F2 generation. Concentration, motility, vitality and morphology were decreased (49.6%, 41%, 36.9% and 26.8%, respectively) in F0 generation. A similar decrement was found in F1 generation (64.6%, 61.9%, 45% and 48.4%, respectively) and in F3 generation (69.1%, 50.6%, 52.4% and 14.6%, respectively) (Table 3) evidencing a transgenerational effect.

**Table 3:** Adult-onset sperm quality parameters over generations.

Generation	n	Group	Concentration (× 10^6^ cells/mL)	Motility (%)	Vitality (%)	Morphology (%)
F0	6	Control	42.67 ± 6.95	57.52 ± 11.15	71.52 ± 4.07	80.33 ± 4.46
F0	6	Exposed	21.50 ± 9.48*	33.92 ± 7.16*	45.12 ± 7.54*	58.83 ± 1.47*
F1	10	Control group offspring	52.00 ± 7.21	53.33 ± 5.77	65.55 ± 5.09	82.38 ± 10.73
F1	10	Exposed group offspring	18.40 ± 4.56*	20.31 ± 8.19*	36.02 ± 3.56*	42.50 ± 16.12*
F2	10	Control group lineage	30.02 ± 6.11	59.06 ± 12.78	67.56 ± 14.41	77.44 ± 10.15
F2	10	Exposed group lineage	19.33 ± 6.43	33.98 ± 7.27	50.18 ± 4.27	58.50 ± 10.83
F3	10	Control group lineage	49.67 ± 2.34	57.713 ± 2.29	67.06 ± 0.50	85.00 ± 7.81
F3	10	Exposed group lineage	15.33 ± 3.06*	28.78 ± 7.60*	31.92 ± 10.87*	72.55 ± 1.88*

(*) Statistically significant differences between control and arsenic exposed group (mean ± standard deviation) compared intra-generationally (F0-F3) (P < 0.05).

### Genotoxic damage

Arsenic can generate genotoxic damage, DNA strand breaks is the most common lesion after generation of reactive oxidative species (ROS/RNS) or by the inhibition of the DNA repair process^18^. DNA fragmentation by arsenic has already been determined in humans^46–49^ and in rodents^50–52^, besides the assessment of DNA breaks in white blood cells (WBCs) by comet assay has been recently proposed as a method for predict risk of disease^53^. DNA fragmentation was evaluated by comet assay in WBC after their membranes and cytoplasm were removed with a lysis solution and subsequent dissolution of the nucleosomes and the unwinding of the DNA supercoil by an alkaline solution treatment in order to expose the alkali labile sites which appear as breaks that migrate towards the anode during electrophoresis producing a ‘comet’-like appearance, with a brightly fluorescent ‘head’ (the nucleus/undamaged DNA) and a ‘tail’ (fragmented DNA) both were recorded as percentages over the generations and were compared intra-generationally between exposed groups and their age-matched control groups.

**Figure 2:**
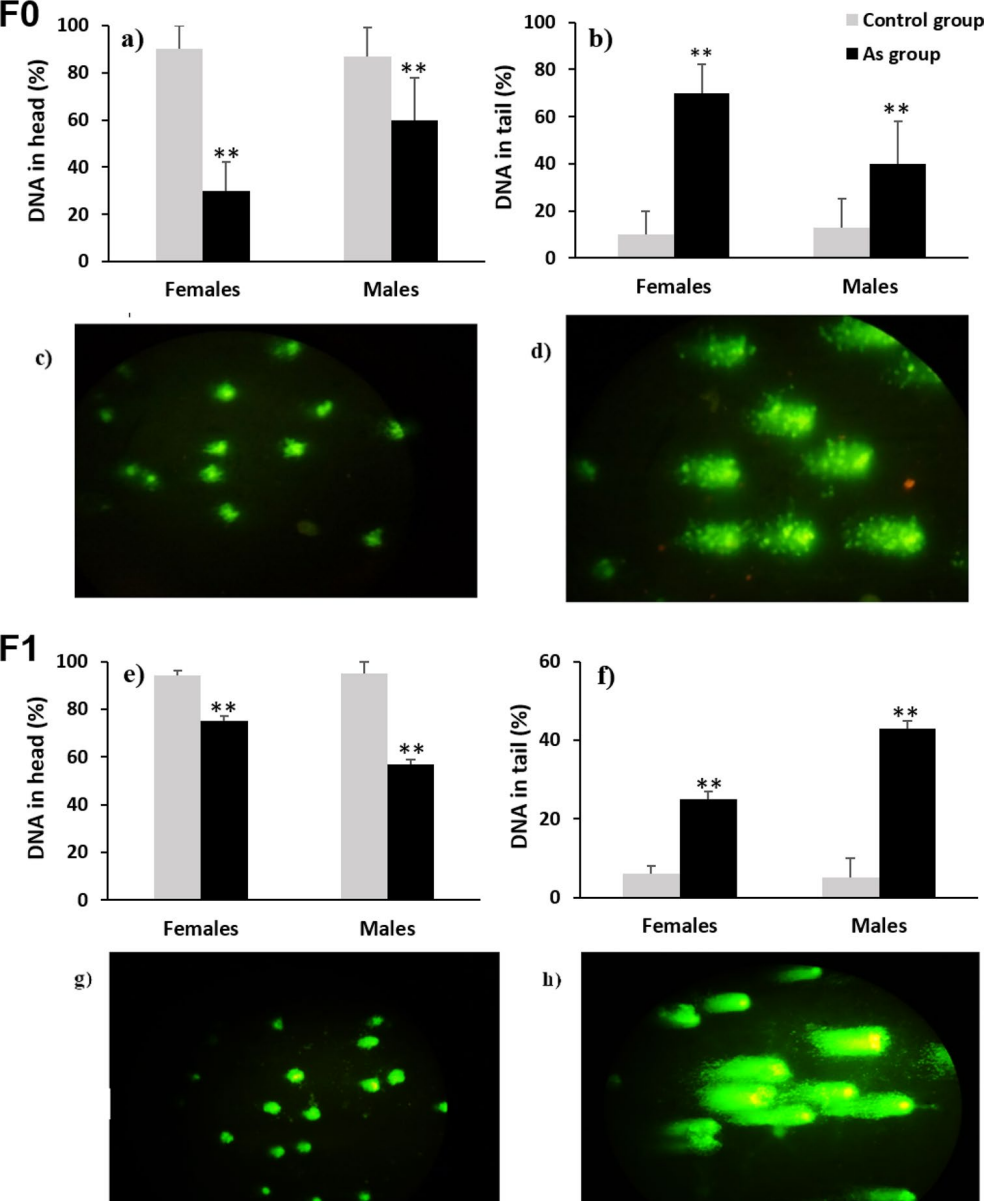
DNA damage in WBC assessed by comet assay (SCGE); (**a**,**e**) show the percentage of DNA in head (integrity) in males and females of the generations F0 (direct exposure) and F1 (partially exposed offspring) respectively; (**b**,**f**) show the percentage of the DNA in tail (fragmentation) in males and females of generations F0 and F1 respectively. Grey bars: control group and linage (n = 24), black bars: As exposed group and linage (n = 24). (*), (**) statistically significant differences intra-gender between control and arsenic exposed group in each generation at p < 0.05 and p < 0.01 respectively. Error bars: standard deviation. (**c**,**g**) microphotographs of WBC without fragmentation (F0 and F1, respectively); (**d**,**h**) microphotographs of WBC with fragmentation (F0 and F1 respectively).

**Figure 3:**
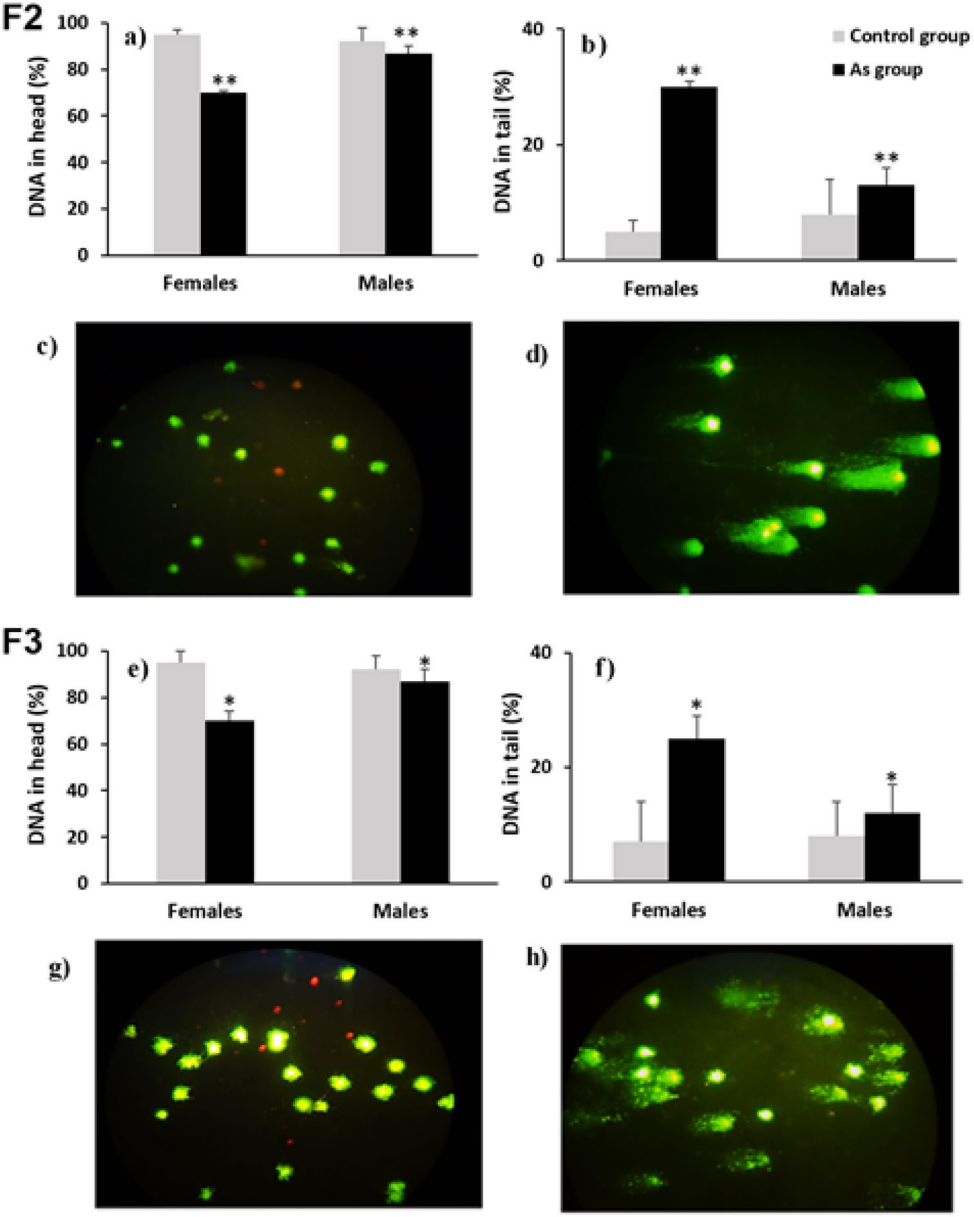
DNA damage in WBC assessed by comet assay (SCGE); (**a**,**e**) show the percentage of DNA in head (integrity) in males and females of the generations F2 (indirect exposed multigenerational lineage) and F3 (transgenerational lineage without partial or indirect exposure) respectively; (**b**,**f**) show the percentage of the DNA in tail (fragmentation) in males and females of generations F2 and F3 respectively. Grey bars: control group and linage (n = 24), black bars: As exposed group and linage (n = 24). (*), (**) statistically significant differences intra-gender between control and arsenic exposed group in each generation at p < 0.05 and p < 0.01 respectively. Error bars: standard deviation. (**c**,**g**) microphotographs of WBC without fragmentation (F2 and F3, respectively); (**d**,**h**) microphotographs of WBC with fragmentation (F2 and F3 respectively).

The comet assay showed a decrement in the DNA integrity (Figs. 2c,g and 3c,g) in the arsenic linage over generations (Figs. 2a,e and 3a,e). There was an increased percentage of DNA in the tail (genotoxic damage; Figs. 2d,h and 3d,h) in females and males (7.0 and 3.1-fold, respectively; Fig. 2b) of the F0 exposed generation fragmentation was also significantly increased in females and males (4.2 and 8.6-fold, respectively; Fig. 2f) of the F1 and in females and males (6 and 1.6-fold, respectively; Fig. 3b) of the F2 generation arsenic lineage. A transgenerational effect was observed in the significant increase in DNA fragmentation in females and males (3.1 and 1.3-fold) of the F3 generation arsenic lineage (Fig. 3f).

### Global DNA methylation in gonadal tissue of F0, F1–F3

Arsenic exposure is associated with epigenetic alterations in different tissues including DNA methylation in blood and testicular tissue^10, 54^. These changes could be observed multigenerationally^19^. Here we determined the percentage of change in global DNA methylation (%Δ5-mC) in the arsenic lineage based on the percentage obtained in the control lineage. In this study statistically significant differences in DNA methylation patterns and in %Δ5-mC were observed in gonadal tissue over generations following the arsenic linage.

**Figure 4:**
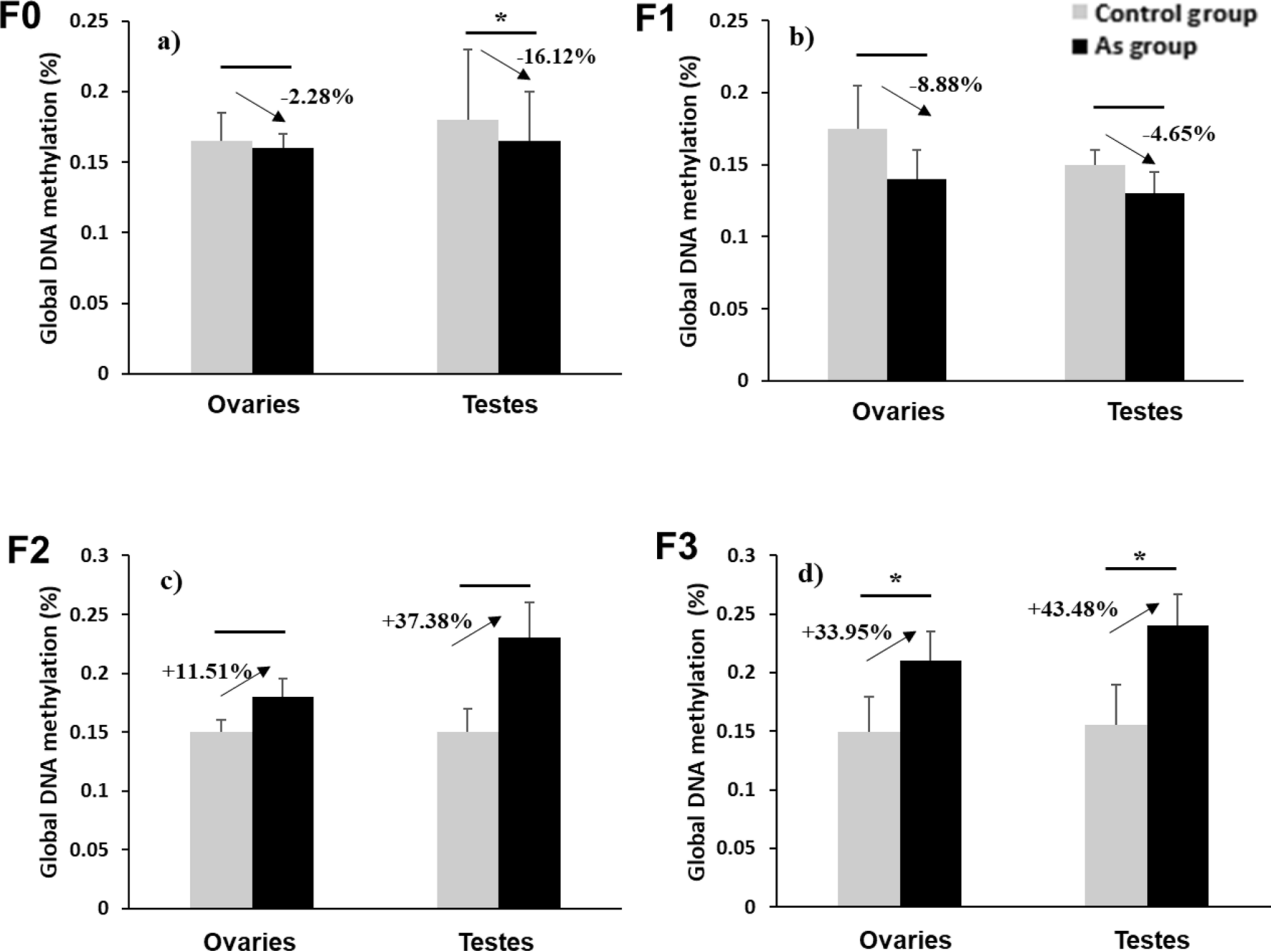
Global DNA methylation and %Δ5-mC (arrows); (**a**–**d**) show intra-gender comparisons between control lineage (n = 24; grey bars) and arsenic lineage (n = 40; black bars) in F0, F1, F2 and F3 respectively. Error bars: standard deviation. (*) p < 0.05.

The methylation analysis showed a hypomethylation (%Δ5-mC = − 2.3%; Fig. 4a) in ovaries and in testes tissue (%Δ5-mC = − 16.2%; Fig. 4a) of the F0 exposed generation; however, only methylation decrease in males was statistically significant. The same pattern was observed in F1 generation arsenic lineage without any statistical significance (Fig. 4b) In contrast, in the F2 and F3 generations of arsenic lineage a significant hypermethylation (%Δ5-mC =  + 37.4%; Fig. 4c) in F2 males and in F3 males (%Δ5-mC =  + 43.5%; Fig. 4d) and females (%Δ5-mC =  + 33.9%; Fig. 4d) were found.

### Histological findings

Arsenic exposure affects the reproductive system including sex organs, such ovaries and testicles in humans and rodents^45^. Morphological aberrant changes in gonads of both females and males after arsenic exposure have been reported^55, 56^. In this study, we evaluated the transgenerational effect of arsenic in gonads using hematoxylin and eosin staining and subsequent histopathological analysis.

**Figure 5:**
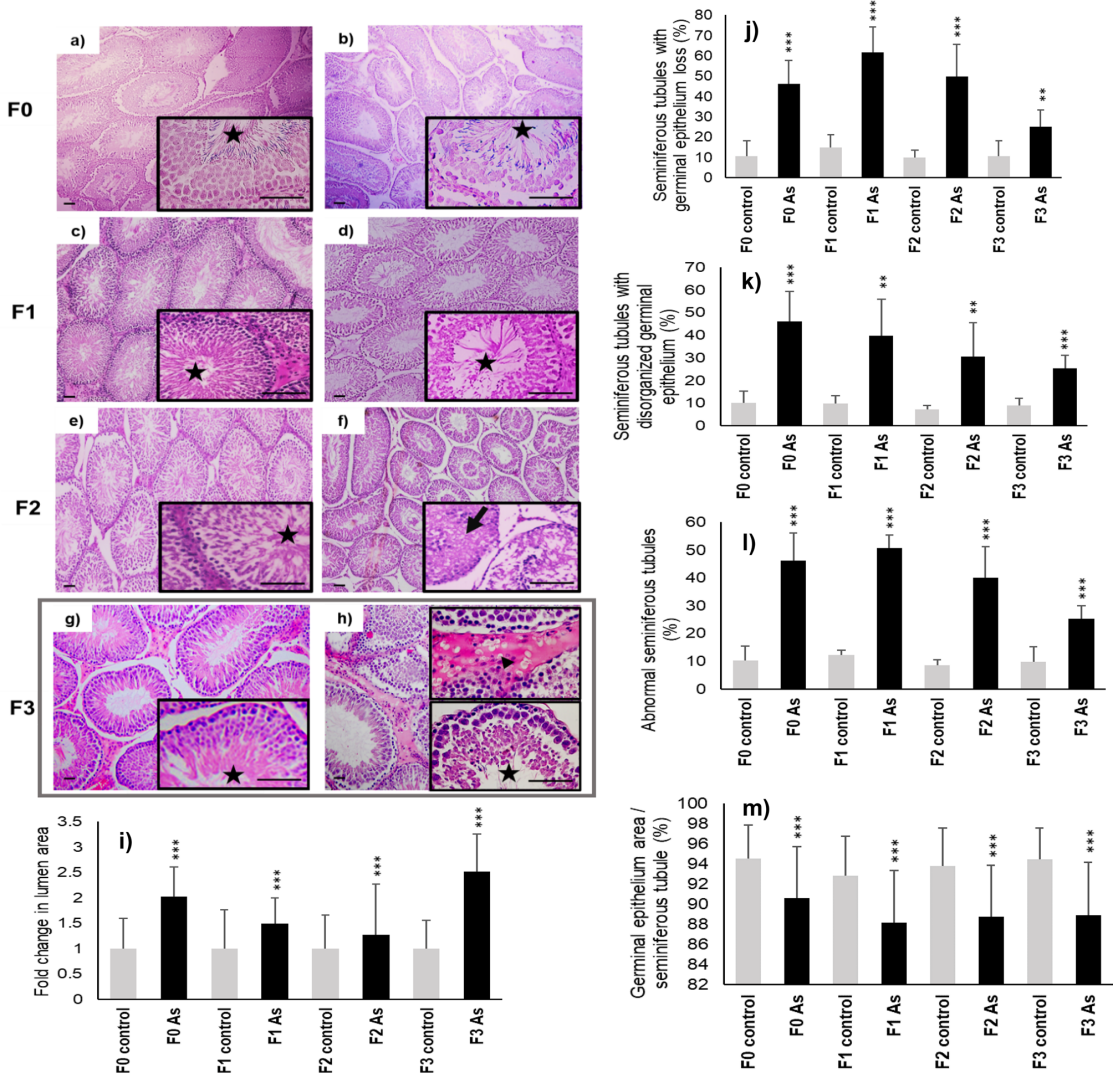
Testicle sections (n = 32), stained with Hematoxylin and Eosin, of males exposed directly to arsenic F0 (**b**), a decrease in sperm cells is observed in the seminiferous tubules (star), a finding that remains constant in subsequent generations (**d**,**f**,**h**); in addition, in the F2 (**f**) generation, tubules with protein material (arrow) were observed and in their descendants, F3 (**h**), abundant vacuoles are visible in the interstitial space (arrowhead). On the other hand, in the control group and their offspring, normal histological characteristics are observed (**a**,**c**,**e**,**g**). Bars = 100 µm. For (**i**) fold change in lumen area, (**j**) seminiferous tubules with germinal epithelium loss, (**k**) seminiferous tubules with disorganized germinal epithelium, (**l**) abnormal seminiferous tubules, (**m**) germinal epithelium area/seminiferous tube values are mean, error bars: standard deviation. (**) P < 0.01, (***) P < 0.001.

**Figure 6:**
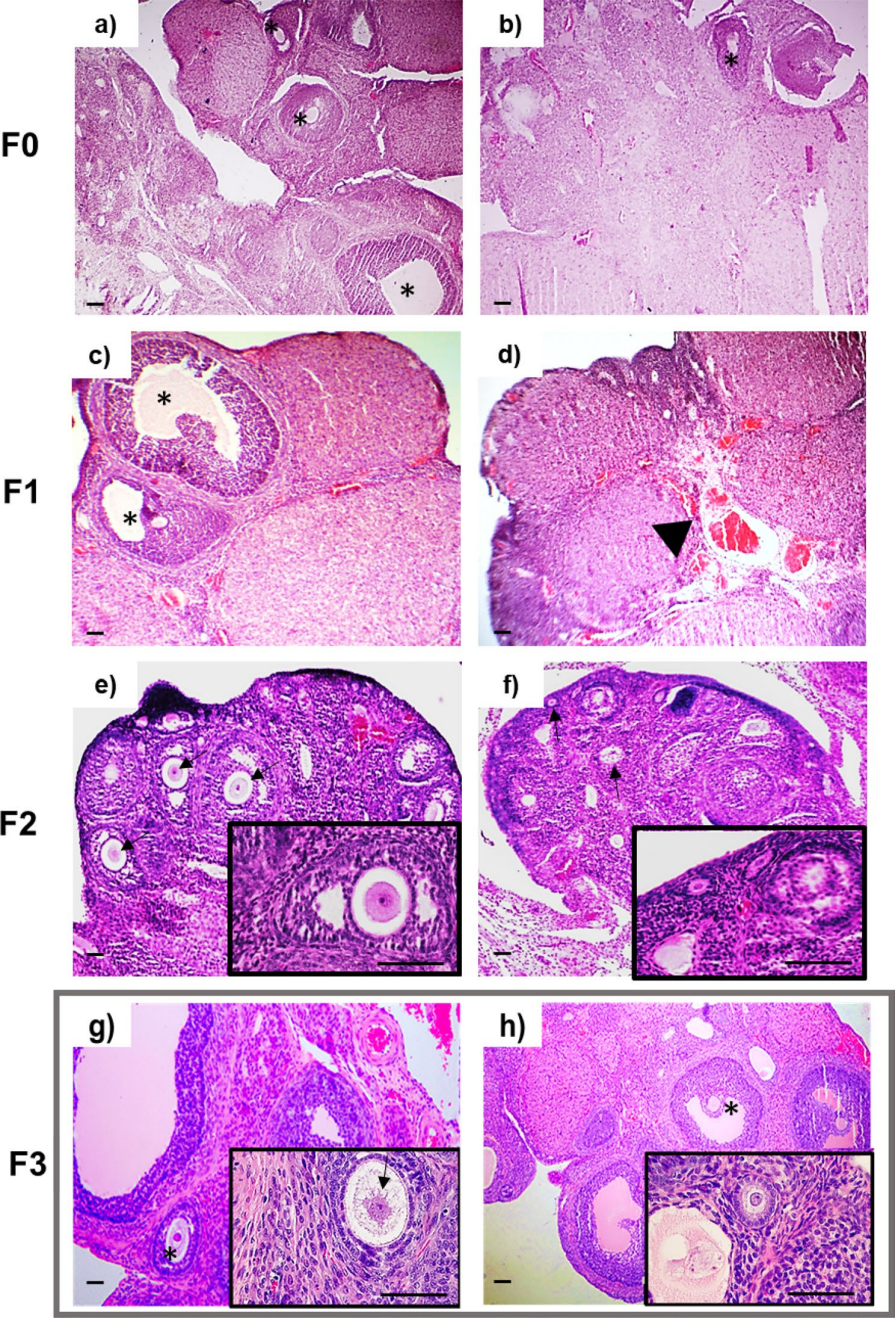
Hematoxylin and eosin stained ovarian sections (n = 32) of females exposed directly to arsenic (**b**) proliferating follicles are observed (*), while in their offspring, F1 (**d**), it's shown marked vascular congestion (arrowhead), the posterior offspring (**f**,**h**) present proliferating follicles with visible ovules (arrows) of normal characteristics. The ovaries of the females in the control group (**a**) and their F1–F3 offspring (**c**,**e**,**g**) presented normal morphological characteristics. Bars = 100 µm.

Gonadal disease was detected in males and females of the arsenic lineage, it was characterized by abnormal changes. In the case of testicular tissue and compared to the control group (Fig. 5a) directly exposed males (F0) presented a lower number of maturing sperm cells (Fig. 5b), a reduction of the epithelium area in the seminiferous tubules (Fig. 5m), augmented seminiferous tubules with germinal epithelium loss (Fig. 5j), disorganization (Fig. 5k), abnormal seminiferous tubules (Fig. 5l) and augmented lumen area (Fig. 5i). In the same way their descent (F1) presented a severe decrease in spermatogenesis with loss of the normal organization of the tubular epithelium (Fig. 5d) compared to the F1 control group (Fig. 5c) including the testicle abnormalities found in F0 (Fig. 5i–m). The pathological findings were constant in the following generations; tubules with proteinaceous material and disorganization of the germinal epithelium (F2; Fig. 5f) compared to the F2 control group (Fig. 5e) and the same testicle abnormalities (Fig. 5i–m) adding the presence of abundant acidophilic vacuoles in the interstitial space in the third generation (F3; Fig. 5h) to the mentioned abnormalities (Fig. 5h–m) in contrast to the F3 control group (Fig. 5g). In the ovarian tissue of the females exposed directly to As (F0) compared to the control group (Fig. 6a), proliferating follicles with slightly hyperchromatic nuclei were observed (Fig. 6b); while the offspring exposed in utero and during lactation (F1) presented a smaller number of proliferating follicles and significant vascular congestion (Fig. 6d) in contrast to the control group (Fig. 6c), on the other hand, in the following two generations (F2, F3) multiple follicles with normal characteristics were found (Fig. 6f,h) compared to the F2 (Fig. 6e) and F3 (Fig. 6g) control groups respectively.

## Discussion

Developmental programming due to early life exposure to environmental influences including toxicants, could generate phenotypical changes in offspring and further generations without any direct exposition, influencing the long-term health of the lineage^3^. The current study demonstrated the transgenerational potential of arsenic exposure to generate phenotypic changes, including those in body weight and different organs, decreased sperm quality, changes in DNA methylation in ovaries and testes tissues, DNA damage in WBC and aberrant gonad morphology, primarily in the testes, in a rat model. Related to body and different organs weight, no significant difference in body weight at birth was found in the arsenic lineage, which contrasts with the toxicological effects of arsenic in the human population, related to low weight and height at birth^34^. Conversely, in some studies with rodents exposed to arsenic during the gestation period, no effect on the body weight of the offspring has been registered^36, 37^. However, it has been observed that even when there are no significant variations in birth weight, it can decrease considerably in postnatal stages^37^. In this study a decrease in body weight in the adult-onset was observed principally in F2 and F3 generations following arsenic exposure. The same effect was observed in females of the F0 generation, showing a direct effect of arsenic mainly on females. Similarly, in a chronic rat model, with exposure to sodium arsenite with 3 times the dose used in this study, adult exposed rats had no statistically significant lower body mass than unexposed rats, being even lower in females than in males, besides, no changes in body weight were observed in the offspring (F1)^57^. A reduction in liver and kidney weight was also observed in females and males of the F0 generation and later in the F2 and F3 generations of the arsenic lineage but only in liver weight. Studies have reported a significant reduction in the weight of the liver^58^ and kidneys^59, 60^ of rats and mice exposed to arsenic. In addition to the effect of arsenic on hepato-renal organ somatic indices, a mechanism that could explain the weight loss is that arsenic can contribute to blockage of cellular glucose uptake due to a carbohydrate depletion, promoting that cellular glucose metabolism cannot be ruled out, besides the diverse mechanisms related to an increased risk of diabetes mellitus in animal models^61^. No significant differences were found in the weight of the testicles in the F0 or F1 generation. However, a significant increase was observed in the F2 and F3 generations of the arsenic lineage. Some studies have reported no change in testis weight^62, 63^ besides a decrease in the epididymis weight^64^ after exposure to arsenic in rats, on the contrary, an increase in testicles and epididymis weight has also been reported^65^; in this study, a decrease in the weight of the epididymis was observed in the F0 generation and an increase and decrease in the F2 and F3 generations, respectively, following the arsenic lineage. As far as we know, there is no evidence of the transgenerational effects of arsenic in the body weight and of various organs, also not related to exposures to other metals, at least in mammals, however, there is evidence related to another environmental toxicants. Exposure to Permethrin and DEET increases the body weight of the offspring in female rats without any change in males, besides a decrease in the weight of epididymis and kidneys in the first generations (F1 and F2) but not in F3^66^. Contrary, phthalates exposure increases the body weight in the first generations with no differences in testicular and body weight in F3^67^. Other studies about exposure to pollutants such as dioxins, DDT and uranium, have found a reduction in body weight of the first generations. In the case of DDT exposition, an increase in body weight and various organs (obesity) was found until the F3 generation^68, 69^.

Exposure to inorganic arsenic has been related to spontaneous abortion, intrauterine fetal death and low birth weight of the offspring, being evidence in both humans and rodents^41, 42^. A reduction in the number of pups per litter over generations was observed. Contrary, studies were individuals were exposed to 10–500 mg/L of arsenic, did not register a significant decrease in offspring but in the sex distribution, decreasing the number of male offspring in the exposed groups to the highest doses^70, 71^. Regarding the sperm quality, we found a persistent decrease (except in F2) through generations. Different studies have reported a decrease in sperm parameters due to direct exposure to arsenic^44, 72, 73^. However, the non-significant reduction presented in the F2 arsenic lineage could be interpreted as an interrupted aberrant phenotype. This interrupted pattern has also been observed in exposures to other toxicants. Parental exposure to benzo(a)pyrene decreased the numbers of seminiferous tubules with elongated spermatids in F0 and F2 but not in F1^74^. Decreased sperm count after prenatal exposure to di- (2-ethylhexyl) phthalate in the F1, F3, and F4 generations but not in F2 was also found. Despite the abnormal morphology of the seminiferous tubules found in this generation^75^. Furthermore, a higher frequency of testicular disease has been detected in the F2 generation due to prenatal exposure to glyphosate but not in F1 or F3 generation^76^. Another study showed an increase in apoptotic testicular cells after exposure to vinclozolin in F1 and F3 except in F2^20^. Interrupted transgenerational phenotypes not related to reproductive aspects such as prostate, kidney and obesity have also been found^21, 76, 77^.

As can affect sperm quality by inducing cell death in testicular germ cells or Sertoli cells^78, 79^ and by decreasing cholesterol metabolism and the production of testosterone. It has been reported that inorganic arsenic (iAs) and its organic derivate DMA can be transferred from the mother through the placenta^36^, generating its toxic effects in the offspring. However, this mechanism would not explain the transgenerational toxic effect by arsenic observed in this study. Epigenetic changes might be involved in the transgenerational inheritance of disease and phenotypic changes including the reproductive toxicity. It has been shown that diverse toxic compounds could exert the potential of promoting altered epigenome in the germinal line, principally DNA methylation patterns and subsequent transmission of aberrant phenotypes across generations in the absence of direct exposure^21, 67, 80–84^. A variety of changes in epigenetic patterns such as DNA methylation, histone posttranslational modifications, and microRNAs have been observed after As exposure in laboratory studies and in human populations^85^. There is a lack of information on the transgenerational effects of exposure to arsenic or heavy metals in general. Most studies are limited to the effects caused in two generations after prenatal exposure^86^.

One of the objectives of this study was the transgenerational evaluation of global DNA methylation in gonadal rat tissue. Based on the mechanisms of arsenic to mediate epigenetic disruption including its own metabolism. Here, different global DNA methylation patterns in both males and females were observed in the arsenic lineage. An hypomethylation in gonads of male and female rats of the F0 generation exposed to arsenic and their offspring (F1) was found. Only the reduction of global DNA methylation in testicular tissue of the F0 generation was statistically significant. The reduction of global methylation in testicular tissue by direct exposure to arsenic has already been reported in mice^54^ in addition to associations between prenatal arsenic exposure and DNA methylation in fetal placenta^87, 88^. Recently, a multigenerational analysis in human population showed that As exposure may leave detectable DNA methylation changes even though exposure occurred decades ago (in grandparents), changes of global DNA methylation were observed among patients afflicted with arsenical skin lesions and differentially methylated DNA loci and regions (744 DML and 15 DMRs) were shared across generations. Besides, a global DNA detection was also assessed in the same study, interesting a not statistically significant hypomethylation for each generation in the arsenic-exposed population compared to the corresponding control population was obtained. Even though 21 DML were hypomethylated and 744 DML had slightly more hypermethylation than in the control groups^19^. Here the same pattern of hypomethylation was found only in F0 and F1 of the arsenic lineage. However, a hypermethylation in F2 and F3 was conserved in both ovarian and testicular tissue of rats from the arsenic lineage, being statistically significant only in the F3 generation. This demonstrates for the first time, the transgenerational potential of arsenic to generate changes in DNA methylation in gonadal tissue of male and female rats. Due to the limitations of the method used in this study for global DNA methylation determinations, we cannot confirm a pattern of transgenerational epigenetic inheritance. However, we hypothesized that the variations in the global methylation profile of the gonadal tissue of rats from the arsenic lineage obtained in this study could be due to intergenerational variations in DML and/or DMRs. Since DMRs of the F1 versus F3 generation sperm cells, were found to be largely distinct after prenatal exposure to an environmental toxin in a laboratory model^19^. Another objective was the evaluation of the transgenerational genotoxicity by the comet assay in WBC, since this assessment has been recently proposed as a method for predict risk of disease in the populations^53^, besides, arsenic is a well-known genotoxic agent^46, 89–91^ that induces its genotoxic effect, including DNA strand breaks, by the resulting oxidative stress after the ROS/RNS generation, during its biotransformation^92, 93^. Here, the results obtained from the comet assays showed a genotoxic effect at a lower dose than that reported in the literature for experiments with chronic exposure of arsenic in drinking water, however, it has been reported that As can induce DNA strand breaks, even at low concentrations^18^. A significant intra-generational increase in the percentage of DNA in tail in the arsenic lineage compared to the control lineage was found in all generations in females and males, however, the inter-genotoxic effect following the arsenic lineage, was in decrement across generations without loss of statistical significance in the F3 generation compared to the control group. These results refer to DNA strand breaks due to the generation of oxidative stress. This genotoxic activity is believed to be one of the main mechanisms by which arsenic exerts its carcinogenic effect^94^. Similarly, chronic exposure of *Snail Physa Acuta* to vinclozolin resulted in genotoxicity, changes in DNA methylation, and in an aberrant reproductive phenotype in F1^95^. Other two-generation (F0–F1) studies have shown an increase in oxidative DNA damage in children exposed to low levels of arsenic in utero and during early childhood^96^. Recently, increased genetic damage in newborns after in utero arsenic exposure assessed by various biomarkers of early genetic effects including DNA strand break evaluated by comet assay was reported, due to an increase in the %DNA in tail^93^. An study in *Daphnia magna* showed that DNA damage (DNA strand breaks) is no longer observed and has no significant impact on the detectable life traits in the (F1–F2) after parental uranium exposure^97^. Nevertheless, no evidence of multi (F2) or transgenerational (F3 and beyond) genotoxic effects after arsenic exposure has been assessed. Here we report a transgenerational genotoxic effect in WBC of rats after a chronical parental exposure to arsenic in drinking water. Litter is demonstrated about the transgenerational effect caused by other environmental toxicants, and the existent evidence about the genotoxic effects over generations don’t show a real transgenerational effect. This is the case of the DNA damage (double strand-breaks and 8-hydroxyguanine) reported in O*ryzias latipes* and caused by chronic exposure to low dose gamma radiation; in this study, the generations (F0-F4) were continuously exposed to the toxicant source^98^, not being possible to establish a real transgenerational effect. We hypothesize that the transgenerational genotoxic effect found in the F3 is due to an hereditably alteration in the antioxidant and/or DNA damage repair systems caused by the parental direct (F0) or the indirect exposure (F1 and F2) experienced by the lineage. This is presupposed based on the evidence of the effects of arsenic on the DNA repair system^48, 99, 100^.

Few two-generation studies related to heavy metals exposure have shown aberrant effects in growth, initial reproduction age and antioxidant capacity in the offspring onset, similarly to the found in the F0. Proposing that the effects found in the first generation are frequently similar to those found in future generations^101, 102^. Interesting, a considerable number of studies have shown environmental toxicants promote the transgenerational inheritance of altered reproductive phenotypes. It has been reported that As exposure caused transgenerational reproductive effects in *C. elegans* might be associated with H3K4 di-methylation and SPR-5 downregulation^33^. However, this has not been explored in mammals until now. Here we demonstrate the negative transgenerational reproductive effects of parental exposure to As_2_O_3_ in drinking water at a concentration of 1 mg/L, through histopathological evaluation of testicles and ovaries in a rat model. The negative effects found in ovarian and testis tissue due to arsenic exposure were constant across generations, at least in males. A decrease in mature spermatocytes (F0) and a disorganization of the germinal epithelial wall of the seminiferous tubules (F1–F3) in addition to a severe spermatogenesis decrease (F1), tubules with proteinaceous material (F2) vascular congestion, acidophilic material and vacuoles in the interstice (F3) was found in males of the arsenic lineage. As exposure can cause atrophy of Leydig cells, reduction of the gametogenic cells and the seminiferous tubules diameter in a dose-dependent way^103^, vacuolization of seminiferous tubular cells, marked reduction in spermatogenesis, interstitial tissue edema, congestion and hemorrhages has also been assessed in the testicular tissue of rats and mice with direct and acute arsenic exposure^56, 104^. Similar results have been observed in the testicles of rats with chronic and prenatal exposure to arsenic^105^. On the other hand, a direct effect was observed in females of the F0 and F1 arsenic lineage. Follicles with hyperchromatic nuclei (F0), a small number of proliferating follicles, and significant vascular congestion (F1) were found. However, no relevant findings were found in the F2 and F3 generations compared to controls. In ovarian tissue, vacuolization of the stroma, atrophic follicles, and pyknotic follicular cells after As exposure have been reported^55, 106^. Toxicant exposition and transgenerational findings related to epimutations in germinal cells include disorganization in the germinal epithelium of mice testis after exposition to Di-(2-ethylhexyl) Phthalate (DEHP) with maintenance of the morphologic abnormalities from the first generation to F3, besides the presence of vacuoles in basal regions. Diminished germ cells in the lumen of seminiferous tubules and increased spermatogenic cell apoptosis have been reported due to DEHP exposition. Transgenerational ovarian cysts but no effect on primordial follicle numbers were detected in females after DEHP exposure^67^. These variations between female and male reproductive phenotypes could be due to variations in arsenic metabolism. Since females have shown higher methylation rates of the inorganic arsenic (iAs), an important process to reduce its toxicity^107^. However, the specific mechanisms by which arsenic might cause these transgenerational phenotypes need to be thoroughly studied. A presumable mechanism of inheritance could be the modified patterns of DNA methylation of the germ cells that could influence the expression of genes involved in DNA repair, this, based in the global patterns found in this study that seems to be modified transgenerationally in the arsenic lineage. Furthermore, we propose that the interrupted patterns and differences in overall DNA methylation observed over generations could be due to DML and/or DMR, because even though toxicant-induced transgenerational aberrant phenotypes could happen, the epimutations linked to these phenotypes can vary inter-generationally. However we cannot discard the genetic mutations that could occur in the germ line, since despite the none or weak mutagenicity reported about this metalloid in vitro, there is evidence about 1.5-fold significant increase in average mutation frequency in liver cells of arsenite-treated mice^108^. So far, no epidemiological studies have reported transgenerational effects in As-exposed populations. This due such long-term studies following more than three generations are hard to be accomplished in humans. Animals models might be a great help to investigate this issue. Despite the arsenic metabolism variations between rats and humans, both seem to present similar toxic effects. Moreover, the model used makes it difficult to differentiate the maternal and paternal contribution to the offspring phenotype. Future studies should be carried out to identify the inheritance pattern of transgenerational adult-onset disease.

We concluded that parental chronic exposure to arsenic (As_2_O_3_) at 1 mg/L has negative transgenerational effects on the adult-onset reproductive phenotype in female and male rats. Besides a genotoxic effect in WBC and an altered global DNA methylation pattern in gonadal tissue which may be linked to the obtained phenotypes. These results indicate that further direct arsenic exposure is not necessary to develop its toxic effects. Combined with recent studies reveal that disturbances in the early life of an individual can affect the health of later generations.

## Methods

### Design and study type

An experimental, prospective, longitudinal and analytical study was carried out, with different litter weaned males (n = 12) and females (n = 12) rats of the Long-Evans stain at about 3 weeks old and with an average weight of 70–76 g. The animals were provided by the bioterium of the Faculty of Medicine at the Autonomous University of Coahuila. The rats were kept under controlled conditions of temperature (21–23 °C), relative humidity (30–70%) and light/dark cycles of 12 h each, with water and food provided ad libitum.

### Experimental groups distribution and exposure

The animals referred above (F0) were randomly divided into 4 groups: male control group (n = 6), female control group (n = 6), male exposed group (n = 6) and female exposed group. Exposed groups received arsenic in drinking water 1 mg/L of arsenic (As_2_O_3_; Sigma Aldrich, St. Luis Missouri, USA) equivalent to 1 ppm, for an interrupted period of 16 weeks, and the control groups received non-As-contaminated water to drink ad libitum. The arsenic dose used in this study was a midpoint based on the highest concentrations of As in drinking water recorded around the world (500–2040 µg/L)^109–111^. Furthermore, this dose of arsenic trioxide has been one of the lowest doses recorded, where reproductive abnormalities are shown in males^38^.

### Breeding

**Figure 7:**
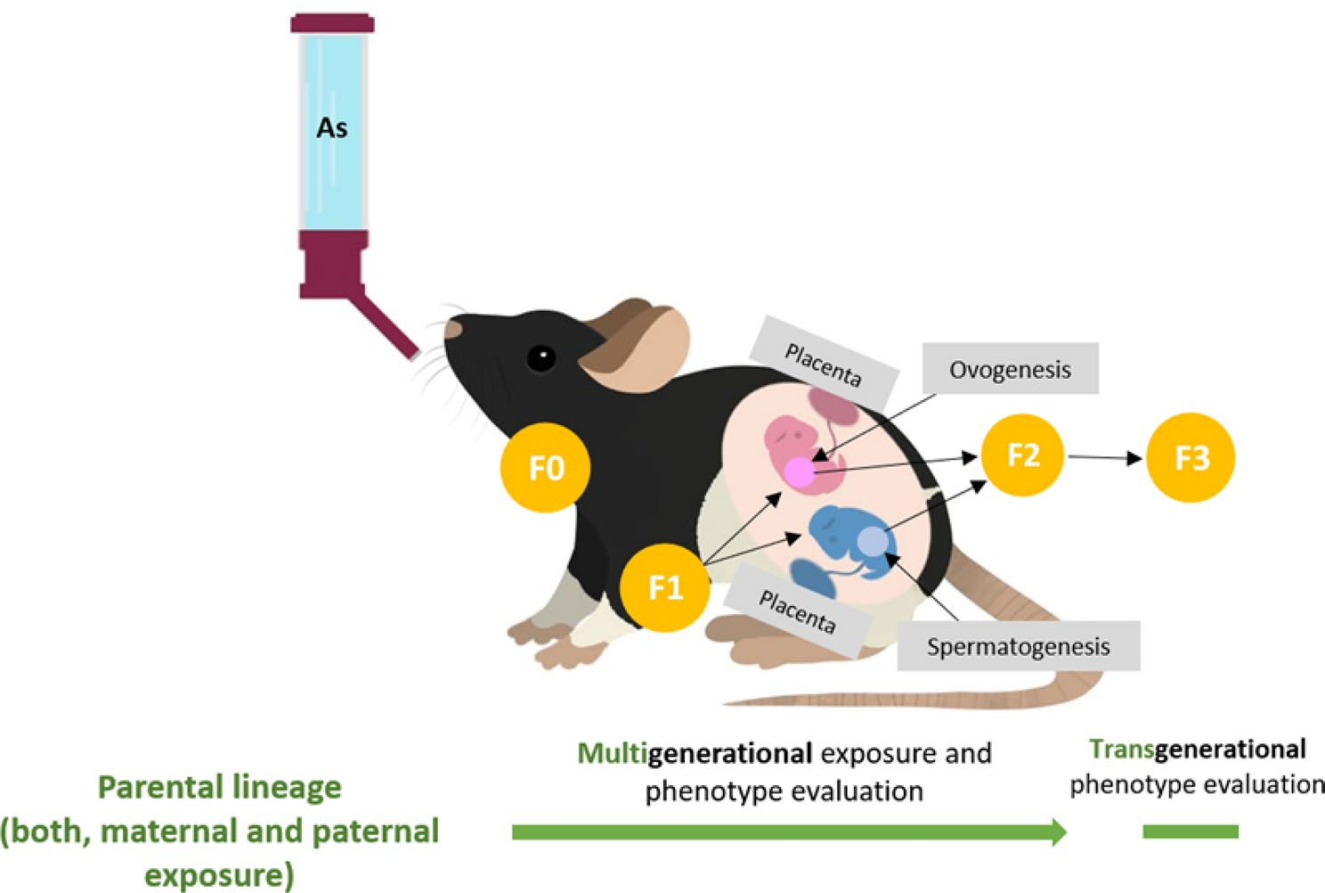
Illustration of the As exposure through drinking water in every generation. *F0* parental direct exposure, *F1* offspring indirect exposure in utero and trough being breastfed until weaning in 21st day, *F2* multigenerational indirect exposure as F1 germinal line, *F3* transgenerational lineage without direct or indirect exposure to arsenic.

To obtain time-pregnant females, randomly chosen females and males rats from F0 control groups were pair-mated in order to obtain control linage. Following 12 weeks (reproductive maturity) of exposure, randomly chosen females and males rats from F0 exposed groups were pair-mated in order to obtain arsenic lineage. After mating and once the copulatory plug was visible, the pregnant females were placed in individual cages until delivery without interruption of the intoxication. The offspring resulting from F0 generation rats were the F1 generation. Non-littermate and randomly chosen females and males from F1 generation of control or As-exposed lineage were bred to obtain F2 generation offspring. F2 generation rats were bred under the same conditions to obtain F3 generation offspring. The type of exposure for each generation is illustrated in Fig. 7. No inbreeding was performed in any generation. Five to ten female and male rats were randomly selected from the arsenic lineage and the age-matched control lineage per generation for subsequent analyzes. A total of 4 (F0-F3) batches of analysis were made.

### Physical evaluation

Rats were weighed with a calibrated analytical balance Tree, basal body weight (at birth) and final body weight (at 4 months of age) were recorded. Besides the body length measure, the number of pups per litter, number of dead pups, and number of pups with malformations were registered.

### Tissue harvest, histology processing and analysis

Once reached adulthood (after four months), rats were sacrificed by cervical dislocation. Liver, kidneys, gonads, and epididymis where dissected and weighed, the results were recorded in relation to body weight (%bw). Total blood samples were collected by cardiac puncture in EDTA coated tubes for the comet assay. About 20 mg of tissue were removed from each gonad (ovaries and testes) and maintained at − 50 °C until DNA extraction. The rest of the tissue and the other organs were fixed in neutral formalin, dehydrated, and embedded in paraffin. 5 μm tissue sections were made and stained with hematoxylin and eosin (H & E) stain. The slides were analyzed by an anatomic pathologist and a morphologist under light microscopy. To quantify the testicular abnormalities, 10 random fields from 5 μm thick sections, using the objective of × 10, were selected. The number of tubules having moderate to severe germinal epithelium loss and disorganization were counted and registered as percentage. The percentage of the germinal epithelium area per seminiferous tubule and of abnormal tubules was determined. Profiles were counted using ImageJ Basics V1.38.

### Collection and evaluation of sperm quality

Sperm samples were collected from the epididymis in tubes with 2 mL of saline solution (NaCl 0.9%) warmed at 37 °C. Sperm parameters: concentration (× 10^6^ sperm/mL), motility (% with progressive mobility), morphology (% normal forms) and viability (% alive) were evaluated after staining, with Vitalsperm (CD MX, Mexico). The evaluation of the samples and duplicates was carried out under light microscopy counting a total of 200 cells per slide. The intra-generational decrease in sperm quality parameters was calculated as: [(average value in the control group—average value in the exposed group)/average value in the control group] × 100.

### Evaluation of genotoxicity in WBC by comet assay

The DNA fragmentation analysis in WBC called single cell gel electrophoresis (SCGE) or comet assay was performed based on the methodology proposed by Singh et al.^112^ and modified by Hartman et al.^113^. 25 μL of whole blood sample was combined with 50 μL of low melting point (LMP) agarose, the volume was pipetted out onto a comet slide (slide coated with medium melting point agarose) which was placed on a flat surface at 4 °C in the dark for 10 min until solidify. The slides were immersed in a lysis buffer solution (2.5 M NaCl, 100 mM EDTA 10 mM Tris Base, 1% Triton and 10% DMSO, pH 10, 4 °C/ 4 h). After lysis, the slides were incubated in a jar containing alkaline buffer (300 mM NaOH, 1 mM EDTA, pH > 13, 4 °C/ 20 min), transferred to an electrophoresis unit with alkaline buffer and subjected to an electric field of 25 V and 300 mA for 20 min at 4 °C. Neutralization was then performed using 0.4 M Tris–HCl, pH 7.5 for 20 min prior to washing with distilled water, then the slides were dehydrated with methanol and allowed to dry. All procedures were carried out under subdued light to minimize possible adventitious DNA damage. The slides were stained with Gel Green, Biotium (CA, USA), observed under a fluorescence microscope (LABOMED Lx 4000) and photographed with Amscope Camera. The fragmentation analysis was done using TriTek CometScore Freeware V1.5 considering parameters such as: percentage of DNA in the tail (fragmentation parameter) and percentage of DNA in the head (integrity parameter).

### DNA isolation and quantification of global DNA methylation

DNA from 20 mg of gonadal tissue (ovaries and testes) was extracted using DNeasy Blood & Tissue Kit of Qiagen (Hilden, Germany) according to the manufacturer’s instructions. The DNA was quantified using NanoDrop (Thermo Fisher) at 260 nm. Purity was determined using 260/280 ratio, only samples with ratio > 1.6 were included. Samples were further diluted with TE buffer at a final concentration of 50 ng/µL. The concentration was later verified using SYBRGreen quantification by fluorescence through a standard curve and using a linear regression model (data not shown).

The quantification of global DNA methylation (%5-mC) was performed using the MethylFlash Methylated DNA Quantification Kit (Epigentek, Farmingdale, NY, USA) following manufacturer’s instructions. Briefly, 100 ng of genomic DNA were used and the 5-mC levels were detected using ELISA-like reaction with a capture antibody, a positive (containing 5% of 5-mC) and negative control (containing 0% of 5-mC) were including. All samples and duplicates were loaded using the same amount of gDNA in the assay plate and the absorbance was measured at 450 nm. The percentage of change in global DNA methylation (%Δ5-mC) was determined as: [(average of DNA methylation value in the exposed group—average of global DNA methylation value in the control group) / average of global DNA methylation value in the control group] * 100. Positive values of %Δ5-mC refer to an increase in the global DNA methylation (hypermethylation) in the arsenic lineage while negative values of %Δ5-mC refer to a decrease in the global DNA methylation (hypermethylation) in the arsenic lineage.

### Statistical analysis

The statistical analysis was performed with the statistical package IBM SPSS Statistics V22.0, descriptive statistics were made, and Shapiro–Wilk was used for the assessment of data distribution. Mann–Whitney U-test and unpaired Student's t-test were applied to determine statistically significant differences between the control lineage and exposed lineage. Differences were considered statistically significant when p < 0.05.

### Ethical aspects

This protocol was proved by the Institutional Committee for Care and Use of Laboratory Animals (CICUAL) (SAGARPA, key code: AUT-B-C-0318–042) of the School of Medicine of the Autonomous University of Coahuila, following the guidelines established in the Official Mexican Standard NOM-062-ZOO-1999, for the production, care and use of laboratory animals. A certified veterinarian supervised the procedures, handling and welfare of experimental animals. The veterinarian was certified by the Secretary of Agriculture, Cattle Raising, Rural Development, Fishing and Food (SAGARPA, key code: MR-0716-33-001-1).
