# Author Correction: Transgenerational effects in DNA methylation, genotoxicity and reproductive phenotype by chronic arsenic exposure

**DOI:** 10.1038/s41598-022-08721-z

**Published:** 2022-03-21

**Authors:** Lydia Enith Nava‑Rivera, Nadia Denys Betancourt‑Martínez, Rodrigo Lozoya‑Martínez, Pilar Carranza‑Rosales, Nancy Elena Guzmán‑Delgado, Irma Edith Carranza‑Torres, Hector Delgado‑Aguirre, José Omar Zambrano‑Ortíz, Javier Morán‑Martínez

**Affiliations:** 1 Departamento de Biología Celular Y Ultraestructura, Centro de Investigación Biomédica, Facultad de Medicina, Universidad Autónoma de Coahuila Unidad Torreón, Gregorio A. García No. 198 sur. Colonia centro, Torreón, Coahuila CP 27000 México; 2 Centro de Investigación Biomédica del Noreste, Instituto Mexicano del Seguro Social, Monterrey, Nuevo León Mexico; 3 División de Investigación en Salud, Unidad Médica de Alta Especialidad, Hospital de Cardiología #34, Instituto Mexicano del Seguro Social, Monterrey, Nuevo León Mexico; 4 Laboratorio de Histocompatibilidad, Unidad Médica de Alta Especialidad (UMAE) # 71, Instituto Mexicano del Seguro Social, Torreón, Coahuila Mexico

Correction to: *Scientific Reports* 10.1038/s41598-021-87677-y, published online 15 April 2021

The original version of this Article contained an error in the Abstract.

“Rats chronically exposed to arsenic in drinking water (1 mg As_2_O_3_/mL) (F0) were mated to produce the arsenic lineage (F1, F2, and F3).”

now reads:

“Rats chronically exposed to arsenic in drinking water (1 mg As_2_O_3_/L) (F0) were mated to produce the arsenic lineage (F1, F2, and F3).”

The original Article has been corrected.

